# CDC Guidance for Community Response to Suicide Clusters, United States, 2024

**DOI:** 10.15585/mmwr.su7302a3

**Published:** 2024-02-29

**Authors:** Asha Z. Ivey-Stephenson, Michael F. Ballesteros, Eva Trinh, Deborah M. Stone, Alexander E. Crosby

**Affiliations:** ^1^Division of Injury Prevention, National Center for Injury Prevention and Control, CDC, Atlanta, Georgia; ^2^Morehouse School of Medicine, Department of Community Health and Preventive Medicine, Atlanta, Georgia

## Abstract

*This is the third of three reports in the MMWR supplement that updates and expands CDC’s guidance for assessing, investigating, and responding to suicide clusters based on current science and public health practice. The first report, *Background and Rationale — CDC Guidance for Communities Assessing, Investigating, and Responding to Suicide Clusters, United States, 2024*, describes an overview of suicide clusters, methods used to develop the supplement guidance, and intended use of the supplement reports. The second report, *CDC Guidance for Community Assessment and Investigation of Suspected Suicide Clusters, United States, 2024*, describes the potential methods, data sources, and analysis that communities can use to identify and confirm suspected suicide clusters and better understand the relevant issues. This report describes how local public health and community leaders can develop a response plan for suicide clusters. Specifically, the steps for responding to a suicide cluster include preparation, direct response, and action for prevention. These steps are not intended to be explicitly adopted but rather adapted into the local context, culture, capacity, circumstances, and needs for each suicide cluster.*

## Introduction

CDC’s National Center for Injury Prevention and Control provides states, local health departments, territories, and tribes with technical assistance for assessing and investigating suspected clusters of suicide and suicidal behavior and responding to suspected and confirmed clusters. Suicide clusters are a group of suicides or suicide attempts that occur closer together in time, space, or both than would normally be observed for a community (*1*,*2*). Clusters might occur in a defined geographic location, called point or spatial-temporal clusters (e.g., part of a school, institution, county, and tribe), or they might be geographically dispersed over long distances (e.g., after a celebrity suicide), called mass or temporal clusters (*3*). This report is focused primarily on point clusters and the response by lead agencies, often state or local health departments or tribal leaders. Responding to suicide clusters is important because, although rare (*4*), these clusters can have devastating immediate and lasting effects on families, friends, and entire communities (*5*).

Definitive confirmation of a suicide cluster can be challenging and is not necessarily a prerequisite for initiating a community response (*6*). If a cluster is suspected (but not confirmed), or if the potential development of a cluster is a concern, the path from assessment to investigation to response is not always linear; steps might occur simultaneously, and a community response might still be appropriate and warranted. In the absence of verification of a cluster, the lead agency, acting from within its authority and in conjunction with community leaders from various sectors (e.g., public health, mental health, health care, education, faith, business, and social services) who have an understanding of the local residents, typically determines whether a response is necessary.

Implementing community-based preventive measures (e.g., programs, practices, and policies) might be appropriate even after just one suicide. Ideally, these preventive measures would already be in place to help prevent suicides. Crisis-response plans implemented in community emergencies, while necessary, are likely not sufficient to address a suicide cluster. State, tribal, local, and territorial public health officials might consider whether and how the specific suicide cluster response plan they create based on the updated guidance can be incorporated into existing plans and practices for emergency management. Consideration might be given to groups who might especially be susceptible to contagion or imitation, such as young persons or persons already struggling with suicidal thoughts (*7*). A suicide cluster or even a single suicide might spur suicide contagion or fear of contagion. Contagion is when the exposure to the suicide or suicidal behavior of one or more persons influences others to attempt suicide (*7*). Although suicide clusters are rare, a suicide might trigger others who are vulnerable to attempt suicide, especially when the death is highly publicized (*8*,*9*). Even if contagion does not occur, clusters can have a profound effect on and create anxiety among community members (*3*); therefore, containing anxiety also might become part of containing the cluster. Suicide also might result in social stigma and feelings of guilt, shame, and anger among family and community members, further increasing the risk for additional suicides (*3*).

Both suicide clusters and suicide attempt clusters can be mitigated by a community response (*3*). However, an important difference exists between the purpose of a response to suicide clusters (to prevent additional deaths) versus suicide attempt clusters (to prevent additional attempts). The guidance presented in this report is applicable to both suicide clusters and clusters of suicide attempts; therefore, both hereafter are referred to as suicide clusters unless otherwise specified.

Previous CDC guidance for responding to suicide clusters was published in 1988 (*1*). Although that guidance is still relevant, this report reorganizes, updates, and expands on that information to include new insights based on current science and public health practice attained using strategies from the original guidance (*10*,*11*). This report provides lead agencies, in conjunction with community leaders, with information on how to best respond to a confirmed or suspected suicide cluster, including strategically tailoring a response plan based on resources, cultural context, and health equity needs of the community. For example, tribal communities might require a response to be tailored to the cultural context of the specific tribe experiencing the cluster. This could involve understanding the history of the tribe, such as population-specific risk factors (e.g., forced relocation, prohibitions on language, and religion), and the recognition of known protective factors (e.g., cultural continuity and the presence of elders) (*12*). This guidance might be revised and expanded as new, pertinent information becomes available.

## Preparing for and Responding to Suicide Clusters

Although suicide clusters are relatively rare events (*4*), lead agencies in conjunction with community leaders can develop a plan for responding (Figure). The general steps of a response plan include preparation, direct response, and action for prevention. Important considerations exist in preparing for and responding to a suicide cluster.

**FIGURE:**
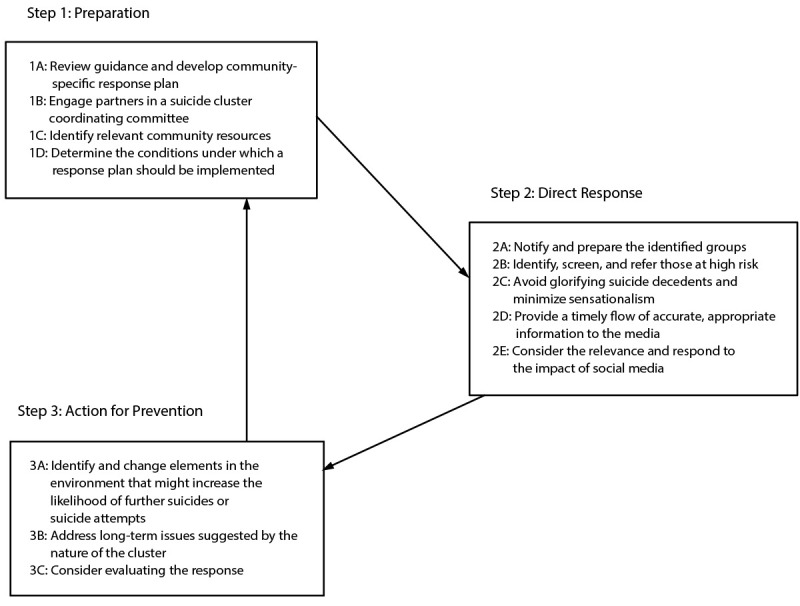
Steps of a community suicide cluster response plan, CDC guidance, 2024

### Step 1: Preparation for Responding to a Suicide Cluster

#### 1A: Review Guidance and Develop a Community-Specific Response Plan

Preplanning can help prevent additional lives lost to suicide. Ideally, the updated guidance should be reviewed before the onset of a suicide cluster. Although not possible in all instances, when faced with a suicide cluster, the community often has an immense sense of urgency that something needs to be done as soon as possible. This sense of urgency can prompt well-intended yet premature actions to be initiated without an agreed-upon community-specific response plan. Certain communities will already have developed a specific response plan. Those that have not can review this guidance and then develop a plan that is tailored to their community. For example, the response plan developed by a tribal community (*12*,*13*) will be different from the response plan developed by a Bhutanese community in the United States (*14*); however, the general steps of a response plan should remain the same (i.e., preparation, direct response, and actions for prevention).

Tribal communities might require a response that can also increase or augment local resources. CDC provided subject matter expert consultation for the Substance Abuse and Mental Health Services Agency’s (SAMHSA’s) publication *Suicide Clusters Within American Indian and Alaska Native Communities: A Review of the Literature and Recommendations* (*12*). CDC also provided subject matter expert review and feedback for SAMHSA’s publication *Preventing and Responding to Suicide Clusters in American Indian and Alaska Native Communities* (*13*). These publications are important resources with specific cultural considerations for tribal communities seeking to prevent and respond to suicide clusters. The overall goal of a response plan remains the same: to contain the cluster and prevent additional deaths and attempts.

#### 1B: Engage Partners in a Suicide Cluster Coordinating Committee

To hear and include perspectives of all concerned sectors of the community (e.g., local government, education, mental health, and public health) that can respond effectively with an all-hands-on-deck approach, representatives from these sectors might be selected by the lead agency to serve for an agreed-upon term (term details can be outlined in the community-specific response plan) on a suicide cluster coordinating committee (committee). Agreed-upon terms can help to retain knowledge, prevent turnover, and provide agency leadership with a time commitment expectation. In the absence of term limits, key points of contact should be made known.

A committee run by a lead agency, often the state or local health department or tribe, is charged with determining when and how to implement a suicide cluster response plan and also might lead or have a large role in investigating potential clusters (*10*). As with many other types of committees the lead agency can designate one staff member to guide the committee through all aspects of the response plan. That person is referred to as the cluster liaison (liaison) and has the following specific response-related duties:

- Convene periodic meetings of the committee to make sure all designated agency representatives are familiar with the community-specific response plan. These meetings can be devoted to assessing whether the response plan is being implemented as intended and revised as needed and taking inventory of changes in the availability of relevant community resources (Step 1C).
- Determine how the liaison will be notified of a suspected suicide cluster. One possible notification mechanism for identifying a suicide attempt cluster at the state or county level might be an alert to the health department by the hospital syndromic surveillance system. Syndromic surveillance is an investigational approach in which health department staff members, assisted by automated data acquisition and generation of statistical alerts, monitor disease indicators in real time or near real time to detect outbreaks of disease earlier than would otherwise be possible with traditional public health methods (*15*). In other instances, potential clusters can be shared with the lead agency by a community member (e.g., a coroner, medical examiner, or school administrator). In this instance, the agency can use an established process to route any notification to the designated liaison while carefully considering any confidentiality and anonymity processes already in place within organizations such as schools and health care systems.
- Soon after notification of a suspected suicide cluster, convene an initial meeting of the committee to review the data and determine whether a community response is warranted (*10*). This cluster and response determination might occur simultaneously.

Although the liaison and lead agency will help the committee navigate through all aspects of the response plan, no one person or agency can or should have to be solely responsible for a community’s response to a suicide cluster. The response plan should be promoted and put into action as a collaborative community effort. In addition to considering sectors to include, the roles needed in the response should be considered so that they are covered as well. For example, communication staff members will be critical to develop and test messages and to ensure consistent safe messaging by all partners. Another important consideration is to enlist the support of at least one locally recognized suicide subject matter expert. If no one is available locally, consider asking a regionally or nationally recognized suicide subject matter expert to serve as a committee consultant. Communities can contact their state suicide prevention coordinator to assist with identifying a potential list of subject matter experts for this role. If new committee members are added, they can be provided with a summary of activities to date along with information about the expectations and goals of the committee.

#### 1C: Identify Relevant Community Resources

Identifying and taking inventory of available resources can help determine a community’s ability or capacity to respond to a suicide cluster and any gaps in community support. Resources can include information that might help contain a suicide cluster (e.g., available crisis and counseling services) and contacts of relevant community agencies. If available, resources also might include funding resources, for example, to support or bolster a local suicide surveillance system or hire and train additional outreach or prevention staff members. Communities lacking these resources or capacities might seek assistance from existing staff members or within the committee agencies with pertinent areas of expertise (Box). Community resources might include school boards, state and local emergency medical services, state and local law enforcement, student and youth organizations (e.g., student government associations, Boy Scouts of America, or Girl Scouts of the USA), and suicide loss survivor groups.

BOX **Potential community resources for a suicide cluster response, CDC guidance, 2024**

- Crisis centers and hotlines
- Local media
- Medical examiners and coroners
- Mental health providers
- Parent organizations (e.g., parent-teacher associations)
- Pastors and ministerial staff members
- School boards
- State and local emergency medical services
- State and local law enforcement
- Student and youth organizations (e.g., student government associations, Boy Scouts of America, and Girl Scouts of the USA)
- Suicide loss survivor groups

The committee can outline and clearly articulate the roles and responsibilities of any community staff member, ideally before the occurrence of a suicide cluster. Many of these staff members will inherently be familiar with how to perform their assigned tasks; however, the committee might also consider and prepare for additional training that might be needed. Subject matter experts from within the identified stakeholder groups might be equipped to offer response-related training and counseling services. Identifying these possibilities early in the planning stages can reduce future costs.

Suicides among young persons are more likely to occur in a cluster than suicides among adults (*3*); therefore, seeking the input of youths can be important. Although not specifically designed for suicide cluster responses, the association of youth-nominated involvement and support (i.e., adolescents nominate caring adults who, with the permission of parents or guardians, learn about ways to support the youths) in helping to reduce mortality among adolescents considering suicide has been documented (*16*). Other opportunities to engage with youths might include reaching out to and including the perspectives of student board members who serve on state suicide commissions or state youth advisory councils.

The input of local media representatives is important when developing the suicide cluster response plan. Leading experts representing organizations that promote suicide prevention, public health, Internet safety, and journalism have developed recommendations for reporting on suicide. When these recommendations are followed, local media could help reduce concerns about contagion, dispel myths and other misinformation, and promote help-seeking behavior. Best practices and recommendations for reporting on suicide were developed by leading experts in suicide prevention and in collaboration with international suicide prevention and public health organizations, key journalists, schools of journalism, media organizations, and Internet safety experts (*17*). Local media can use and tailor these best practices and recommendations to be even more specific and relevant to a grieving community. Journalists often can best convey the message of the importance of these best practices and recommendations to their peers.

#### 1D: Determine the Conditions Under Which a Response Plan Should Be Implemented

The ultimate decision regarding whether and when to implement the cluster response plan lies with the committee. Communities can consider the following conditions when making this determination:

- A suicide cluster has been either confirmed or is suspected within a community (*10*). Because cluster identification is not always clearly delineated, each community should determine the threshold for what is considered higher than expected morbidity and mortality and therefore unusual for their community. If a cluster is suspected, whether the number of cases reaches a predetermined statistical significance might not matter (*6*). A community response plan should be implemented to prevent additional suicides and suicide attempts.
- When suicide contagion is a concern (*18*). If the committee is concerned or determines that the exposure to the suicide or suicidal behavior of one or more persons influences others to attempt suicide (*19*), a response plan should be implemented.
- A common-source issue has been identified. For example, the Great Recession and other historical economic downturns have been associated with increases in suicide rates (*20*). The committee might determine that an increase in suicidal behavior and suicides among persons within a community experiencing foreclosure and housing displacement (*21*) is a suicide cluster and constitutes the need for a community response.

Ultimately, no definitive formula exists for determining whether and when to implement a cluster response plan. However, each community can rely on the committee for the final decision.

### Step 2: Direct Response to the Cluster

#### 2A: Notify and Prepare the Identified Groups

Step 2 begins with notifying the various staff member resources identified in Step 1C. Immediate notification of a suicide cluster among staff members who have integral roles in the crisis response is important. Although certain staff members might already be aware of the cluster, others might not, and it is ideal for those who are unaware to receive initial notification and accurate information directly from the committee rather than finding out via rumors potentially on social media or receiving the news from another outside source. The liaison and the committee members have shared responsibility that should be strategically divided. For example, the committee representative for the department of education might lead the contacting, notifying, and preparing of staff members within affected school systems (as outlined in that community’s specific response plan) while carefully considering any confidentiality or anonymity processes already in place. Although different committee representatives will deliver the message to various groups, making sure that the messaging is the same, per the preparation step, is important. Coordinated communication and consistent messaging is a vital part of the response because it can help to prevent confusion and create community cohesion.

Next, the various groups identified should be prepared by refamiliarizing staff members of their roles and responsibilities in the context of responding to a suicide cluster. An important part of staff member preparation is considering and troubleshooting potentially stressful scenarios including any shame and guilt associated with the occurrence of additional suicides along with the realities of staff member burnout and need for self-care (e.g., make counseling services available to staff members).

#### 2B: Identify, Screen, and Refer Those at High Risk

An important part of a community response to a suicide cluster is to identify, screen, and refer to services persons in the community who might be at increased risk (*22*). The committee might first consider identifying the following persons who could be at increased risk:

- Persons close to the decedents, including
  º relatives of the decedents (e.g., parents, children, and siblings),
  º boyfriends, girlfriends, or partners (current and former) and other close friends of the decedents, and
  º close work colleagues or fellow students of the decedents
- Persons with previous exposure to the suicide or suicides or other heightened risk factors, including
  º persons with lived experience who have attempted or thought about suicide,
  º persons with a history of depression or other mental illness or with co-occurring mental illness,
  º persons who are known to isolate or who lack social support, and
  º persons with other known risk factors for suicide (more information about risk and protective factors for suicide is available at https://www.cdc.gov/suicide/factors/index.html)

The response will vary depending on whether the cluster is among youths or adults. For adult clusters associated with a particular occupation or workplace, consider asking supervisors, colleagues, and other employees about who might be at increased risk. For youth clusters, the committee might consider asking teachers and students for support with identifying youths who might be at increased risk. Another consideration is the ability for a trained counselor to conduct a screening interview with persons who might be at increased risk for suicide. For youths, identification, screening, and referral to services might be provided by school resources because of legal considerations and the need for parental consent. Although a parent’s consent to have their children screened can override a youth’s refusal to assent, both adults and youths who are at increased risk sometimes do not want to be screened in fear of involuntary mental health services. Making information regarding the 24 hours/7 days a week availability of the national 988 Suicide & Crisis Lifeline (call or text 988 or go to https://988lifeline.org) available to all persons provides those who do not want to be screened as part of the cluster response with an important and alternative touchpoint for help.

Regardless of whether the suicide cluster is among youths or adults, another important consideration for screening and referrals is the local availability of community mental health and other services. Counseling support and mental health services might look different for different communities depending on the availability of staffing resources. The increase in demand for counseling services does not always align with an increase in the number of staff members needed to support a community surge in referrals in response to a suicide cluster. The committee will need to determine the types and availability of support resources for identifying persons at high risk for suicide (often completed as part of the first preparatory step). As general measures of and support for identifying persons at high risk, the committee might take the following actions:

- Make counselors available at various community hubs (e.g., local YMCAs, churches, and schools) and consider offering walk-ins and taking appointments.
- Get the support of local media and social media influencers to call public attention to the availability of counselors and other sources of support including the national 988 Suicide & Crisis Lifeline.
- Use social media and other online forums as tools to communicate with community members, including those potentially at high risk, about available services and support.

#### 2C: Avoid Glorifying Suicide Decedents and Minimize Sensationalism

Any glorification of persons who died by suicide and sensationalism when messaging about the death might increase the risk for suicide among those who might be thinking about or who have attempted suicide in the past, have other risk factors, or both (*8*). Glorifying is defined as praising, worshipping, or bestowing honor and admiration (*23*). Sensationalism, often referenced in journalism, is defined as the use of exciting or shocking stories or language at the expense of accuracy to provoke public interest or excitement (*24*). When the community plans to celebrate the life of, pay tribute to, or honor a person who has died by suicide, the consequences of intentional and unintentional glorification and sensationalism should be considered. Similarly, avoiding defamation of the decedents can prevent unnecessary pain and additional grief to their families along with social isolation among those who might relate to them. Therefore, the committee can help the community determine the response.

Memorials for persons who died by suicide often are well intended but can have serious, unintended consequences (*25*). The committee might need to provide resources and education to the community for navigating and helping to manage this fine line between celebrating a life and glorification and sensationalism. Media recommendations for reporting on suicide emphasize this response step (i.e., avoid glorifying and sensationalism); however, this guidance is also needed among the community at large in response to a suicide cluster.

Considerations for the committee representatives include the following:

- When the deceased is a student, consider privately notifying the students closest to the person. Then proceed with notifying the remaining student body in closely supervised small groups.
- Provide maximum support to community members when announcing the suicide of a loved one. This might vary by the availability of resources. In one community, maximum support might allow for the hiring of additional school counselors, social workers, or psychologists. For another community, maximum support might involve temporarily partnering with faith-based organizations and workplace employee assistance programs to repurpose staff counselors as trained crisis counselors. If local and in-person resources are limited, consider setting up and making telehealth options available.
- Provide community groups and members (e.g., students, teachers, school administrators, families, and media) with facts that provide a holistic and as exact a picture as possible of the person who died by suicide.

#### 2D: Provide a Timely Flow of Accurate, Appropriate Information to the Media

The media has a substantial impact on when and how the public is informed of a suicide cluster and can have a major role in how public attitudes and opinions are formed. As a result, providing the media with timely information that is both accurate and appropriate can help keep the public informed. Including a local media representative in the development of a community response plan has value. Including the local media will help facilitate the delivery of safe messaging from the media while the committee maintains their ability to respond.

Considerations for the committee include the following:

- Assign a media spokesperson to each committee community sector (e.g., education, public health, and local government). These designees need not be the same as the sector representatives identified in Step 1B; however, in smaller communities with limited staff members and resources, the same representatives might serve in both capacities (i.e., same person is both sector representative and media spokesperson). Additional media designees for community groups identified in Step 1C also can be assigned, as needed.
- Assign one media information coordinator whose primary role is to ensure that the messaging is the same across all media spokespersons. This person’s responsibilities include regular communication delivering appropriate, approved, and up-to-date information with all spokespersons; fielding media inquiries to the appropriate media spokesperson; compiling and updating a resource list of national and local subject matter experts to properly triage media requests; arranging and hosting press briefings; avoiding the downplay of the crisis, which could weaken the authority of the designated media spokesperson and community leaders; and ensuring that all spokespersons have media training and are knowledgeable in recommendations for reporting on suicide. Safe and consistent messaging is vital to the response and helps to prevent confusion and create community cohesion.
- Request the community’s assistance in directing all media requests for information to the designated media spokesperson. Consider making this information available to community members via an informational pamphlet (if time and resources allow) or as a social media post by community leaders.

#### 2E: Consider the Relevance and Respond to the Impact of Social Media

The original guidance developed by CDC in 1988 on responding to suicide clusters (*1*) predated the Internet and social media. Since that time, these technological advances have served as both a suicide cluster response tool and a potential risk to the response (*26*); however, applicability will vary by community. For example, rural communities might not have access to reliable Internet service and, therefore, the use of social media among community members might be limited. For other communities with regular access to the Internet, the relevance and impact of social media on a suicide cluster response might need to be considered.

##### Social Media as a Response Tool for Prevention and Intervention

Social media can serve as a helpful response tool for the prevention and intervention of suicide clusters. Social media can be a forum for communities to talk about suicide and suicide clusters safely (*26*,*27*). The social media platforms that are being used by the affected communities should be identified. The following are considerations for using social media as a response tool:

- Committee representatives can use social media to disperse accurate and appropriate information (ensure content is mobile friendly) to community members about a past or present suicide cluster. This can include providing
  º facts that debunk myths about suicide and any rumors or misinformation about the suicide cluster,
  º prevention-related (*28*) and positive messaging including stories of hope and recovery (*29*),
  º information and direct links to the national 988 Suicide & Crisis Lifeline (messaging about the Lifeline, including on social media, resulted in an increase in calls and a reduction in suicide) (*28*,*30*),
  º information about local suicide prevention and mental health community resources, and
  º opportunities for committee representatives to answer questions and address concerns of community members in near real time.
- Certain social media companies have consulted with suicide subject matter experts to assist with developing protocols for identifying and supporting those at risk for suicide (*26*). The committee can consider the following points:
  º Certain social media platforms allow friends to flag someone who might be at risk (*26*). The flag alerts platform staff members who can virtually push various prevention messages and information about available suicide prevention resources directly to the persons of concern (*26*).
  º Research has debunked the perception that more sensationalized articles shared via social media are more appealing to readers. News articles that are responsibly and safely reported were more likely to be shared on social media (*31*).
  º Social media and other online forums can serve as tools to communicate with community members, including those potentially at high risk for suicide, about available services and support (Step 2B).
  º Social media has been used as a prevention and response tool with various age groups and populations (*26*) and also can be used to analyze communication patterns during a suicide cluster (*32*).

##### Social Media as a Response Risk to Prevention and Intervention

Social media can serve as a risk to the suicide cluster response. For certain communities, social media can be a forum for persons to talk about suicide and suicide clusters irresponsibly and therefore has the potential to have a negative impact on those at increased risk for suicide. The following are considerations for social media as a response risk:

- Social media can serve as an unwelcome early notification system of a suicide or suicide cluster. The speed at which information travels via social media can prompt the spread of misinformation among community members and contagion among vulnerable persons. As a result, community leaders often are left to deal with the traumatic aftermath of many persons having to first learn of a loved one’s death by suicide online rather than carefully and thoughtfully by the process set forth in Steps 2A and 2B.
- Social media might be used to seek peer support as opposed to professional help.
- Social media might be used as a forum to glorify the decedent and sensationalize the death. Any glorification of persons who died by suicide and sensationalism when messaging about the deaths might increase the risk for suicide among those who are most vulnerable.
- Social media can serve as an unmoderated forum where posts are often not able to be redacted (e.g., live streaming).

### Step 3: Action to Help Prevent the Next Cluster

#### 3A: Identify and Change Elements in the Environment that Might Increase the Likelihood of Further Suicides or Suicide Attempts

Identifying and changing environmental factors that have the potential to make suicides or suicide attempts more likely is an important part of preventing suicide clusters. Suicide is caused by multiple factors acting at the individual, relational, community, and societal levels. Therefore, prevention that addresses not only individual-level change (e.g., increased help seeking) but that also focuses on changes to the environment can have great effect. CDC’s Suicide Prevention Resource for Action includes the best available evidence for suicide prevention for persons and across communities (*33*).

Creating protective environments seeks to improve settings where persons live, work, learn, and play. This can include reducing access to lethal means among persons at risk for suicide through safe storage of firearms and medications as well as policies (e.g., waiting periods to purchase firearms) that increase the time between thoughts of suicide and the decision to act. In addition, creating protective environments can include intervening at suicide hot spots or locations where persons are known to die by suicide through creation of physical barriers on bridges and tall buildings, installation of call boxes, or posting of signage with a hotline number. Creating protective environments also might include organizational policies in the workplace or within institutions (e.g., jails and prisons) to promote help seeking and other adaptive norms, raise awareness of signs for suicide risk, and support persons who need immediate care (*33*).

Finally, creating protective environments might include other community-based policies such as those that seek to reduce excessive alcohol and substance use by reducing the number of places that sell alcohol in an area. Such measures can reduce not only suicide risk but risk for community and interpersonal violence (*33*). When creating this component of the response strategy, the committee should take a wide range of potentially pertinent environmental elements into consideration.

#### 3B: Address Long-Term Issues Suggested by the Nature of the Cluster

Although many issues are potentially associated with suicide clusters, long-term issues that are specific or unique to the cluster are important to identify and address. Common factors and precipitating circumstances often are identified among the suicide decedents as part of the assessment and investigation of a cluster (*10*). Incorporating the use of surveillance systems such as the National Violent Death Reporting System (https://www.cdc.gov/violenceprevention/datasources/nvdrs/index.html) can allow communities to have a more comprehensive picture of the circumstances surrounding the cluster deaths. These data can empower communities with knowledge about commonalities such as a higher or lower prevalence of relationship, financial, or substance use problems. Although a previous mental health diagnosis was identified as a precipitating circumstance for many decedents, more than half of National Violent Death Reporting System decedents who died by suicide did not have a known mental health condition, emphasizing the importance of other precipitating circumstances and long-term issues (e.g., relationship problems or loss, recent or impending crises, and life stressors) (*34*).

Long-term concerns suggested by the nature of the suicide cluster often can provide an opportunity for communities to engage in more focused upstream suicide prevention efforts. Upstream suicide prevention focuses on factors that influence the likelihood that youths and adults will become suicidal (i.e., before the emergence of suicidal behavior) (*35*). Community investments upstream can help to prevent future clusters and suicides in general. Long-term issues might exist within various levels of the social ecological model, which emphasizes the interconnectedness among individual, relational, community, and societal levels (*36*).

One long-term issue that often plagues communities at the societal level of the social ecological model is the stigma surrounding suicide. This stigma often creates a roadblock to help seeking and accessing suicide care (*33*) and trickles down to all other social ecological levels, including the community level. To address stigma in communities, it must first be acknowledged. A community’s ability to address stigma requires time, patience, and resources.

#### 3C: Consider Evaluating the Response

Although multiple evidence-based strategies and approaches exist that effectively decrease suicide morbidity and mortality (*33*), less evidence is available regarding strategies and approaches for responding to suicide clusters. When feasible, conducting a postresponse evaluation, similar to a disaster response after action report (*37*), could be considered to build best practices to support future responses. Evaluation will be important for growing the literature on responding to suicide clusters while simultaneously helping communities to be better prepared for future clusters.

The committee can consider how to incorporate (and which are most appropriate to include) evaluation indicators into future responses. Even if a formal evaluation is not feasible, at a minimum, future response plans can be updated in response to the community’s feedback on how effectively the current plan worked. Aspects of the response plan that worked can be retained and parts that did not work or were not applicable can be removed for an updated version of the plan. Although the response to a suicide cluster might end with this step, communities should return to Step 1 in preparation for future clusters.

## Conclusion

CDC guidance regarding the steps for responding to a suicide cluster are designed to empower communities with guidance to help tailor and direct their response plans. CDC’s vision is no lives lost to suicide. To advance this vision, CDC is using data, science, and partnerships and the best available evidence to prevent suicide, with a focus on upstream prevention as well as interventions designed to lessen the immediate and long-term harms associated with suicide and suicide attempts. This comprehensive approach implemented in communities across the United States can help save lives (https://www.cdc.gov/suicide).
